# Deep Learning in Endoscopic Ultrasound: A Breakthrough in Detecting Distal Cholangiocarcinoma

**DOI:** 10.3390/cancers16223792

**Published:** 2024-11-11

**Authors:** Rares Ilie Orzan, Delia Santa, Noemi Lorenzovici, Thomas Andrei Zareczky, Cristina Pojoga, Renata Agoston, Eva-Henrietta Dulf, Andrada Seicean

**Affiliations:** 1 3rd Department of Internal Medicine, Iuliu Hațieganu University of Medicine and Pharmacy, Victor Babeș Str., No. 8, 400012 Cluj-Napoca, Romania; 2 Regional Institute of Gastroenterology and Hepatology, Croitorilor Str., No. 19-21, 400162 Cluj-Napoca, Romania; cristinapojoga@yahoo.com; 3 Automation Department, Faculty of Automation and Computer Science, Technical University of Cluj-Napoca, G. Baritiu Str., No. 26-28, 400027 Cluj-Napoca, Romanianoemi.lorenzovici@gmail.com (N.L.);; 4 Department of Clinical Psychology and Psychotherapy, Babeș-Bolyai University, Sindicatelor Str., No. 7, 400029 Cluj-Napoca, Romania; 5 Faculty of Medicine, Iuliu Hațieganu University of Medicine and Pharmacy, Victor Babes Str., No. 8, 400012 Cluj-Napoca, Romania

**Keywords:** bile duct cancer, cholangiocarcinoma, endosonography, artificial intelligence

## Abstract

Introduction: Cholangiocarcinoma (CCA) is a highly lethal malignancy originating in the bile ducts, often diagnosed late with poor prognosis. Differentiating benign from malignant biliary tumors remains challenging, necessitating advanced diagnostic techniques. Objective: This study aims to enhance the diagnostic accuracy of endoscopic ultrasound (EUS) for distal cholangiocarcinoma (dCCA) using advanced convolutional neural networks (CCNs) for the classification and segmentation of EUS images, specifically targeting dCCAs, the pancreas, and the bile duct. Materials and Methods: In this retrospective study, EUS images from patients diagnosed with dCCA via biopsy and an EUS-identified bile duct tumor were evaluated. A custom CNN was developed for classification, trained on 156 EUS images. To enhance the model’s robustness, image augmentation techniques were applied, generating a total of 1248 images. For tumor and organ segmentation, the DeepLabv3+ network with ResNet50 architecture was utilized, employing Tversky loss to manage unbalanced classes. Performance evaluation included metrics such as accuracy, sensitivity, specificity, and Intersection over Union (IoU). These methods were implemented in collaboration with the ADAPTED Research Group at the Technical University of Cluj-Napoca. Results: The classification model achieved a high accuracy of 97.82%, with precision and specificity both at 100% and sensitivity at 94.44%. The segmentation models for the pancreas and bile duct demonstrated global accuracies of 84% and 90%, respectively, with robust IoU scores indicating good overlap between predicted and actual contours. The application performed better than the UNet model, particularly in generalization and boundary delineation. Conclusions: This study demonstrates the significant potential of AI in EUS imaging for dCCA, presenting a robust tool that enhances diagnostic accuracy and efficiency. The developed MATLAB application serves as a valuable aid for medical professionals, facilitating informed decision-making and improving patient outcomes in the diagnosis of cholangiocarcinoma and related pathologies.

## Simple Summary

Cholangiocarcinoma (CCA), a rare cancer that affects the bile ducts, is often diagnosed at a late stage, making treatment difficult and survival rates low. This research aims to improve the accuracy of diagnosing distal cholangiocarcinoma (dCCA) by using advanced artificial intelligence (AI) models to analyze endoscopic ultrasound (EUS) images. By developing a user-friendly tool, this study hopes to assist doctors in making quicker and more reliable diagnoses, potentially leading to better outcomes for patients with this challenging disease.

## 1. Introduction

Cholangiocarcinoma (CCA) is a malignancy originating in the intrahepatic or extrahepatic bile ducts, characterized by its late diagnosis and high mortality rates. Although relatively rare, CCA is the second most common primary liver tumor and the most common biliary malignancy, constituting approximately 3% of all gastrointestinal neoplasms [1]. CCA includes intrahepatic, perihilar (pCCA), and distal subtypes (dCCA), each associated with high recurrence rates post-surgery and a 5-year survival rate ranging from 10 to 40% [2]. Despite advances in endoscopic techniques, differentiating benign from malignant biliary strictures remains a formidable challenge, particularly in the early stages of cholangiocarcinoma [3].

Endoscopic ultrasound-guided fine-needle aspiration (EUS-FNA) offers a powerful tool for CCA detection, combining high-resolution imaging with cytological sampling of the biliary system and adjacent structures. EUS-FNA has demonstrated superior sensitivity and accuracy compared to other diagnostic techniques, positioning it as a preferred method for CCA diagnosis [4]. However, mastering endoscopic ultrasound (EUS) requires an extensive learning curve, necessitating the integration of both cognitive and technical skills. Expertise in EUS is typically acquired through repetitive practice at specialized training centers, given its limited availability. Furthermore, the declining number of specialized pathologists, coupled with increasing diagnostic demands, underscores the importance of both time efficiency and diagnostic accuracy [5,6]. Additionally, EUS-FNA has a low negative predictive value, meaning a negative result does not definitively exclude malignancy [7,8,9].

Recent advances in image processing have significantly impacted medical research and clinical practice. Artificial intelligence (AI) has wide-range applications in gastrointestinal endoscopy, such as the detection of colon polyps [10,11], pancreatic cancer [12,13], and the assessment of the tumoral invasion depth [14]. Automating image analysis through advanced deep learning and AI techniques offers a promising solution to reduce diagnosis time, increase accuracy, and extend diagnostic capabilities to regions with limited access to medical experts [15]. Deep learning, a subset of machine learning, focuses on learning data representations rather than relying on specific algorithms, thereby broadening the practical applications of AI [16].

A pivotal advantage of deep learning methods is their ability to automatically learn representations of entities, thus eliminating the need for time-consuming manual design. Deep learning techniques have been successfully applied to image recognition, object detection, and speech recognition [17]. The rising popularity of Convolutional Neural Networks (CNNs) has significantly fueled interest in deep learning. With high-quality training data and image preprocessing tailored to the specifics of cholangiocarcinoma, CNNs could effectively differentiate between benign and malignant biliary tumors [12]. CNNs consist of multiple convolutional layers that automatically extract relevant features from images. Each layer is specialized to detect particular features such as edges, colors, textures, or pixels by applying filters to the input image to create feature maps. Pooling layers reduce the size of these feature maps, improving the information stability and reducing sensitivity to minor distortions. Max pooling and average pooling are the most common techniques used in this process.

However, there are few reports addressing the application of AI in EUS for dCCA detection, particularly focusing on the biliary system rather than the pancreas. This research pioneers the development of a custom AI model using advanced CNNs to automatically detect dCCA from EUS scans, a relatively underexplored area in AI applications. Beyond merely detecting tumors, our model uniquely contours the pancreas and bile duct, which adds an innovative segmentation capability that was absent in previous studies. This dual-functionality approach significantly enhances diagnostic precision, streamlines workflow, and reduces the likelihood of false-positive or false-negative results, thereby improving patient outcomes. By addressing the current gap in biliary imaging applications of AI, this study contributes new insights and methodologies to the growing body of literature on AI-assisted diagnostics in gastroenterology.

This article is organized as follows: The Materials and Methods section outlines patient selection, EUS image acquisition, and the development of the CNN and DeepLabv3+ networks for dCCA detection and segmentation. The Results section presents performance metrics, including accuracy, precision, and sensitivity. The Discussion section addresses the clinical significance of these findings and their implications for AI in EUS diagnostics. Finally, the Conclusion summarizes the study’s key contributions and future directions for AI applications in gastroenterology.

## 2. Materials and Methods

### 2.1. Patients

This retrospective study was conducted at the Regional Institute of Gastroenterology and Hepatology Cluj-Napoca. The study protocol adhered to the guidelines of the 1975 Declaration of Helsinki and received approval from the ethics committee (approval no. 7750, dated 30 May 2024). EUS images from consecutive patients diagnosed with dCCA were recruited from March 2023 to July 2024. The inclusion criteria were (a) the presence of a distal bile duct tumor identified on EUS, with biopsy confirmation of cholangiocarcinoma by a pathologist, (b) age 18 years or older, and (c) the ability to provide written consent. The exclusion criteria were (a) history of previous treatment for hepatobiliary tumors, (b) prior biliopancreatic surgery, (c) patient refusal to participate, and (d) duodenal stenosis impeding full examination of the common bile duct.

### 2.2. Procedure

For the EUS examinations, a linear echoendoscope (Olympus GF-UCT 180 AL5; Olympus, Tokyo, Japan) was used in conjunction with an ultrasound platform (Hitachi ARIETTA 850). Compared to the radial EUS, the linear approach offers enhanced visualization from the hepatic portal region to the superior bile duct, providing better detail of the branching areas of the celiac and superior mesenteric arteries. Second, EUS-guided biliary puncture is conducted using linear EUS, whereas radial EUS is limited to diagnostic purposes only [18]. All procedures were performed by two experienced gastroenterologists (AS, CP) with over 1000 EUS-FNA and more than 200 CH-EUS procedures each.

### 2.3. Preprocessing Images

A total of 162 EUS images were initially collected from patients diagnosed with dCCA. However, after careful evaluation, only 112 of these images depicted dCCA, while 50 represented a healthy pancreas. Among the dCCA images, 106 were deemed suitable for use in the study. Some images were excluded due to difficulties in accurately labeling the tumor regions with the imageLabeler tool in MATLAB, as the tumor boundaries were not distinct enough. This decision was made to prevent inaccuracies in the model’s performance and ensure the integrity of the dataset used for training. Each image was stored in a database as both an original and a copy. The copies were labeled by the medical experts to demarcate the pancreas, bile duct, and tumoral mass in the images with tumors and the pancreas and bile duct in the healthy images. This labeling facilitated the training of the artificial intelligence (AI) system in recognizing tumors.

**Figure 1:**
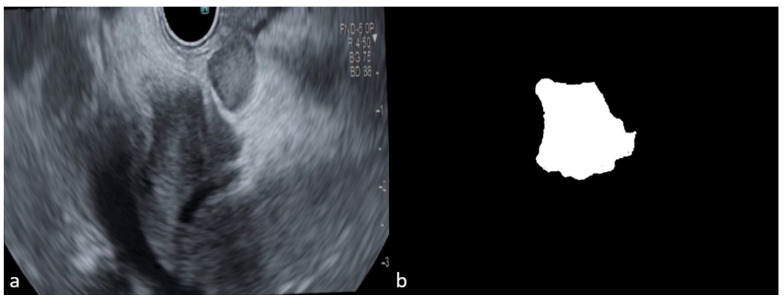
(**a**) EUS aspect of a dCCA; (**b**) binary mask for tumor.

Given the limited size of the dataset, image augmentation was necessary to increase its diversity and ensure the model could handle the variability of real-life images. Augmentation techniques applied included random rotations between −30 and +30 degrees, horizontal mirroring, random translations on the X and Y axes up to +/− 10 pixels, random scaling between 0.9 and 1.1 times the original size, and adding Gaussian noise with mean 0 and variance 0.01. Each original image was augmented to create approximately eight additional copies, each with one of these techniques applied randomly. This process resulted in 848 images containing tumors and 400 images with a healthy pancreas. To ensure compatibility with the model’s input requirements, all images and corresponding labels were resized to 256 × 256 pixels. This uniform resizing enabled the training of the convolutional neural network (CNN), prevented errors due to varying image sizes, and ensured consistency across the dataset. Training the CNN for detection and segmentation required both the original images and binary masks corresponding to the labeled organs. These masks, created from the labeled images, denoted the tumor, pancreas, and bile duct regions. An example of an image and its corresponding mask is presented in Figure 1.

For efficient data management, the images were split into two files with suggestive names. The images were read from these files and automatically labeled based on the filename. Using the “imageDatastore” function, images containing tumors and those with a healthy pancreas were combined into a single dataset. The dataset was subsequently split into training, validation, and test sets, with this division carried out based on images. It is specified that all images in the test set were original; 80% of the data were allocated for training, 15% for validation, and the remaining 5% were reserved for testing. This structured process ensured accurate reading and labeling of data and an efficient division of images to train and validate the CNN model.

### 2.4. Designing the CNN for Classification

**Figure 2:**
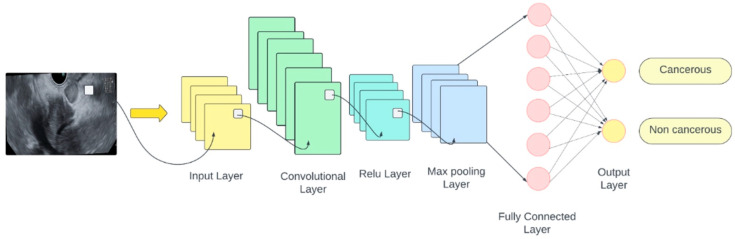
Architecture of the CNNs.

Given the benefits introduced in medical diagnostics by CNNs, the present research chose to develop a custom CNN for our classification model based on the data available [19]. The network architecture was carefully designed to optimally adapt to the characteristics of the dataset, and it was defined by specifying a list of layers to extract features of interest from each image. The input size for the images was set to 256 × 256 pixels with three color channels (RGB space). Convolutional filters were applied to extract features, followed by normalization through the batchNormalizationLayer function to speed up training, stabilize the network, and prevent saturation. A max-pooling layer with a 2 × 2 pooling window size was used to reduce the feature map size. The fully connected layer, fullyConnectedLayer, had two classes: cancer and normal. An output normalization layer, softmaxLayer, was applied to represent the classification probabilities. The final classification layer, classificationLayer, represented the network’s output. The structure of the convolutional neural network architecture, including all the layers used for the classification model, is shown in Figure 2.

After defining the architecture, the training parameters were set. The ADAM (Adaptive Moment Estimation) optimization algorithm was chosen due to its high efficiency and performance in training CNNs and its widespread support in most machine learning libraries [20]. Compared to other optimization algorithms like SGD (Stochastic Gradient Descent), which has a fixed learning rate and can become slow and unstable, or AdaGrad, which adapts various learning rates but becomes very slow during training, ADAM was preferred for its speed and suitability for handling large volumes of medical imaging data [20]. The training options were set as follows: 20 epochs, a mini-batch size of 32, validation every 5 epochs, and progress display during training.

### 2.5. Designing the DeepLabv3+ Network for Detection and Segmentation

**Figure 3:**
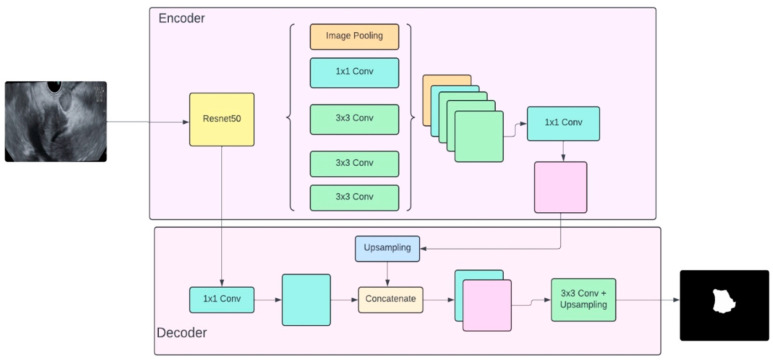
DeepLabv3+ architecture based on Resnet50.

Detection and segmentation of medical images require a more sophisticated convolutional neural network (CNN) than classification. While a customized CNN suffices for classification, DeepLabv3+ was selected for segmentation due to its advanced capabilities. ResNet50, a deep learning network with 50 layers, employs residual connections to prevent performance degradation in data-intensive networks. This characteristic makes ResNet50 highly effective in extracting relevant features from medical images, which is essential for accurate tumor edge, pixel color, and texture segmentation. Figure 3 illustrates the DeepLabv3+ architecture, incorporating ResNet50, and includes both the encoder and decoder components.

The network training process begins with two datasets: original EUS images and corresponding binary masks indicating tumor locations, created through manual labeling. These datasets are combined into a unified datastore, ensuring simultaneous image and label analysis during training. The combined dataset is divided into three subsets: 80% for training, 15% for validation, and 5% for testing. This division ensures comprehensive model training and evaluation. The DeepLabv3+ was customized to fit the dataset’s specific needs. Input images were set to a size of 256 × 256 pixels in grayscale. The number of classes is defined as two: “tumor” and “background.” The initial layers of the standard DeepLabv3+ architecture were modified to better fit the dataset. A new input layer, “newInputLayer”, was defined for images sized 256 × 256 × 1, ensuring the model accepts correctly sized images without additional resizing during training. The initial convolution layer was replaced with “newConvLayer”, matching the kernel size to the input data. For segmentation, the Tversky Loss function was employed due to its effectiveness in handling unbalanced classes. This function, governed by parameters “alpha” and “beta”, balances false positives and false negatives, optimizing tumor detection accuracy. A false positive in this context occurs when a non-tumor region is incorrectly identified as part of the tumor, while a false negative occurs when an actual tumor region is not detected. The training parameters were set to optimize model performance. The “Adam” optimizer, known for its efficiency in medical image processing, was used with an initial learning rate of 1 × 10^−4^. The training process included a maximum of 20 epochs to control duration and prevent overfitting. The mini-batch size was set to 16, and training progress was displayed every 20 mini-batches. Following the same steps as in the development of the tumor segmentation model, binary masks were created for surrounding pancreatic tissue and the bile duct, and two separate networks were trained, one for each organ. Both networks were based on the same DeepLabv3+ architecture, but the class names were adapted to reflect the specific organ being segmented.

Finally, the network was trained using the prepared training and validation datasets. This comprehensive setup ensures the network is well-prepared for accurate tumor detection and segmentation in endoscopic ultrasound images.

### 2.6. Performance Measures for Testing and Validating Classification and Segmentation Models

Testing and validation are crucial stages for refining and improving the performance of models. Performance metrics provide objective evaluations of how well the models perform classification and segmentation tasks, identifying strengths and weaknesses effectively. To evaluate the classification model, which distinguishes between cancerous and healthy images, the following metrics were used: Accuracy, Precision, Specificity, and Sensitivity. For evaluating the segmentation model, which identifies and delineates tumors, the following metrics were employed: Global Accuracy [21], Mean Accuracy [22], Mean Intersection over Union (IoU) [23], Weighted Intersection over Union (Weighted IoU) [24], and Mean Boundary F1 Score [25]. These metrics ensure a thorough assessment of both classification and segmentation models, guiding enhancements to achieve high accuracy and reliability in medical diagnostics. The novel algorithm was developed by researchers from the ADAPTED Research Group, part of the Energy Transition Research Center at the Technical University of Cluj-Napoca.

## 3. Results

### 3.1. Classification and Segmentation Model Performance

**Figure 4:**
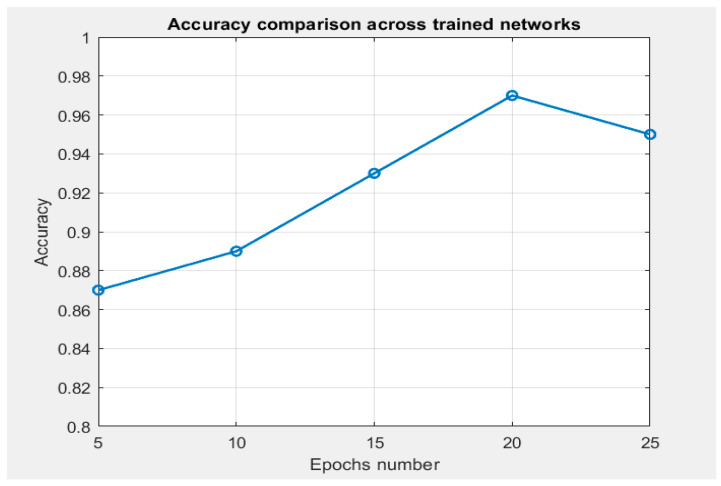
Evolution of accuracy based on the number of epochs for tumor identification.

The initial model developed was the classification model, which is essential for determining whether the input image contains cancer. Only if the image tests positive for cancer will the process proceed to tumor segmentation. The classification and segmentation model report results for tumor identification are available in Table 1. For the pancreas and bile duct segmentation model, performance metrics are available in Table 2. The classification model underwent a series of epoch adjustments to identify optimal performance settings. Initially, the model was trained with 5 epochs, and this number was progressively increased up to 25 epochs to analyze performance. After these trainings, the accuracy achieved in each case was tracked, as shown in Figure 4.

**Figure 5:**
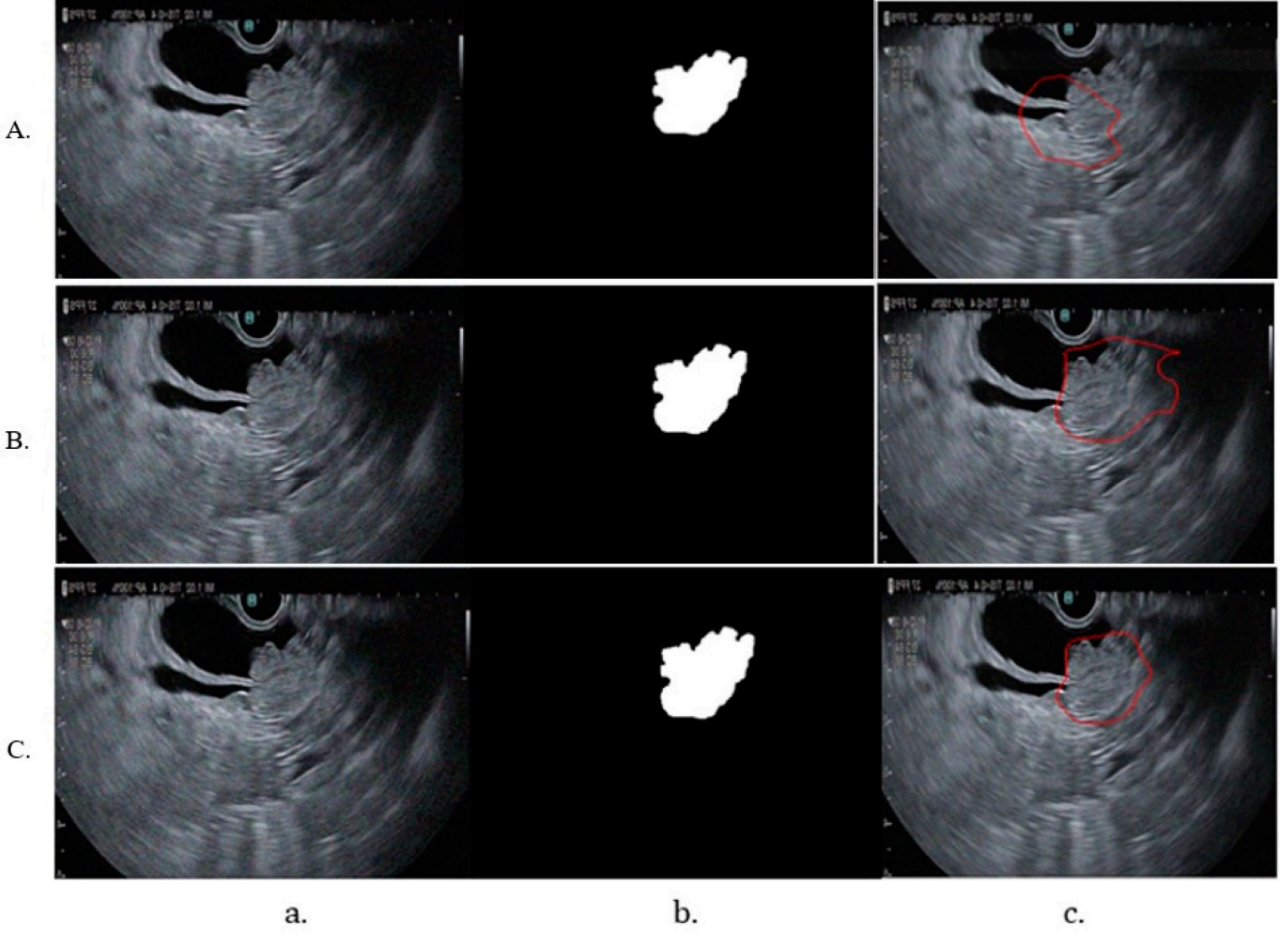
The different stages of tumor segmentation models after repeated training. (**A**) Tumor detection after the first training; (**B**) tumor detection after adjusting the brightness and contrast of the images to highlight the contours; (**C**) final training focusing on the alpha and beta loss parameters within the Tversky Loss function; (**a**) original image; (**b**) binary mask; (**c**) testing image.

**Figure 6:**
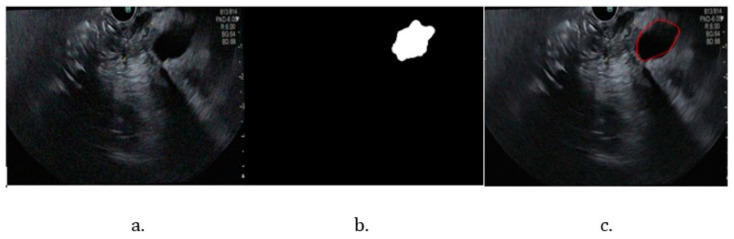
Bile duct pathway segmentation. (**a**) Original image; (**b**) binary mask; (**c**) testing image.

The repeated training and the different stages of tumor segmentation models are represented in Figure 5. The bile duct segmentation model-generated contour over the original image is shown in Figure 6.

### 3.2. Comparing the Efficiency of Segmentation Models

**Figure 7:**
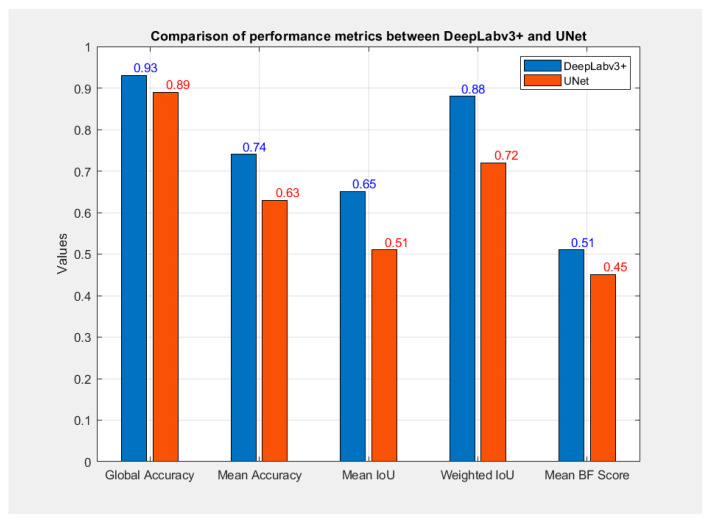
Comparison of performance metrics between DeepLabv3+ and Unet.

**Figure 8:**
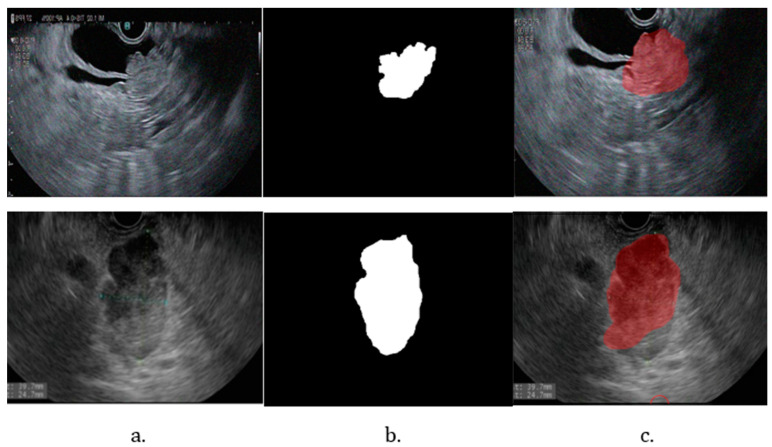
Results of tumor segmentation using UNet. (**a**) Original image; (**b**) binary mask; (**c**) testing image.

Validation of the model’s performance was achieved by comparing the CNN model with a UNet model implemented in Python. The comparative metrics, illustrated in Figure 7, demonstrate the evaluation of both models’ effectiveness in segmentation tasks. Results of tumor segmentation using Unet are depicted in Figure 8.

## 4. Discussions

Regarding dCCA diagnosis, the classification model achieved an impressive accuracy of 97.82%, demonstrating its high efficiency and robustness in correctly classifying nearly all cases. Notably, the model’s precision and specificity both reached 100%, indicating that all cases identified as positive were indeed accurate, with no false positives or negatives. The sensitivity of 94.44% further underscores the model’s strong capability in accurately detecting positive cases. Collectively, these results suggest that our classification model is a reliable and powerful tool for identifying cancerous lesions in EUS images. For the detection and segmentation model, a high Global Accuracy of 93.42% was observed, reflecting a strong overall measure of segmentation correctness across the entire image. The Mean Accuracy of 74.05% indicates balanced performance across different classes, though it also highlights potential challenges with certain categories. The Mean IoU reflects a good overlap between the model’s segmented regions and the ground truth segmentations, while the high Weighted IoU value demonstrates the model’s effective handling of less frequent classes, showcasing its strength in managing unbalanced class distributions. However, the Mean Boundary F1 Score of 51.06% points to the need for improvement in accurately delineating object boundaries, as this metric is particularly sensitive to edge precision. Overall, while the segmentation model shows robust performance, with commendable Global and Weighted IoU scores, there remains an opportunity for refinement in enhancing the accuracy of boundary delineation, which is crucial for clinical applications.

The optimal performance settings were identified as 20 epochs, which yielded the highest accuracy of 97.82%. Beyond this point, the model began to overfit, as indicated by the decrease in accuracy observed when training with 25 epochs. This decline in performance on the validation dataset, despite continued improvement on the training dataset, underscores that the model was fitting too closely to the training data and was losing its ability to generalize effectively to new data.

During training, significant fluctuations in the learning process on the training dataset were observed. To stabilize this process, the learning rate was reduced, allowing for a more gradual convergence towards an optimal solution. The segmentation model underwent multiple training iterations to enhance its efficiency in detecting and segmenting EUS images. Initial analyses revealed that the model identified only 50% of the tumor region (Figure 5A), indicating difficulty in accurately learning tumor contours. To address this, a function to adjust the brightness and contrast of the images was introduced, enhancing contour visibility during training. This function was applied randomly to various images, ensuring that some had their brightness adjusted while others had their contrast modified, with both parameters set to 0.5 to highlight contours effectively. As illustrated in Figure 5B, the introduction of this function improved the detection of the tumor region. More training sessions were conducted to optimize the model’s performance, focusing particularly on adjusting the alpha and beta parameters within the Tversky Loss function. Systematic testing of these parameters revealed that the configuration with alpha = 0.3 and beta = 0.7 yielded the best results (Figure 5C), as it effectively penalized false negatives, leading to improved tumor detection and segmentation.

The performance metrics for the pancreas segmentation model (Table 2) demonstrate good performance, with high accuracy and reasonable overlap between the model-generated contour and the actual contour of the pancreas. However, the Mean Boundary F1 Score indicates the need for further optimization to improve contour precision. For the bile duct segmentation model, the Mean Boundary F1 Score of 45.68% suggests that the model has difficulty accurately segmenting the contours of the bile duct.

To gain a comprehensive understanding of the performance of the MATLAB-implemented model, a comparison with a UNet model implemented in Python was conducted. The performance comparison between the two models reveals that the MATLAB-implemented model outperforms the UNet model in most metrics, as depicted in Figure 7. Although the differences are not substantial, the UNet model currently requires enhancements to match the performance levels achieved by the DeepLabv3+ network, as shown in Figure 8. The MATLAB-implemented model exhibits superior generalization on the dataset, suggesting it is well-trained and resilient to variations in the test set. Furthermore, with a higher Mean Boundary F1 Score, the DeepLabv3+ demonstrates an improved capability to detect and accurately delineate object boundaries.

To our knowledge, only one study addresses EUS imaging for bile duct annotation. Yao et al. developed an AI-assisted linear EUS system that can recognize standard bile duct scanning stations, provide operational instructions to physicians, segment the bile duct with high precision, and automatically measure bile duct diameter. The system’s accuracy surpassed that of senior EUS endoscopists and matched EUS experts, although it was validated only in an augmentation reading study and did not include lesion identification functionality [26].

Despite the limited studies specifically addressing EUS for cholangiocarcinoma, there are notable studies into the application of AI in cholangioscopy. AI algorithms, such as CNN-cholangioscopy, can significantly improve diagnostics for suspected malignant biliary strictures or masses. High accuracy in AI-based imaging allows patients with suspicious lesions on EUS or cholangioscopic images to proceed to surgery even if biopsy results are negative for malignancy [27]. Saraiva et al. developed a CNN to differentiate between normal/benign and malignant cholangioscopy images based on histopathologic evidence, achieving an overall accuracy of 94.9%, with a sensitivity of 94.7%, specificity of 92.1%, and an area under the curve (AUC) of 0.988. The rapid image processing capability of the CNN suggests its suitability for real-time clinical application, potentially streamlining the diagnostic workflow and improving patient outcomes [28]. Other studies, including those by Ghandour et al. and Ribeiro et al., demonstrated high sensitivity and specificity in detecting abnormal cholangioscopic features and papillary projections, respectively [29,30].

**Figure 9:**
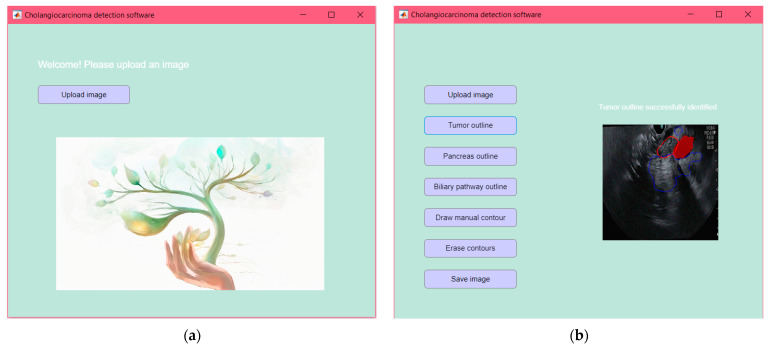
(**a**) Main interface of the novel CCN used for dCCA detection; (**b**) the user interface has various functionalities for analyzing endoscopic ultrasound images.

**Figure 10:**
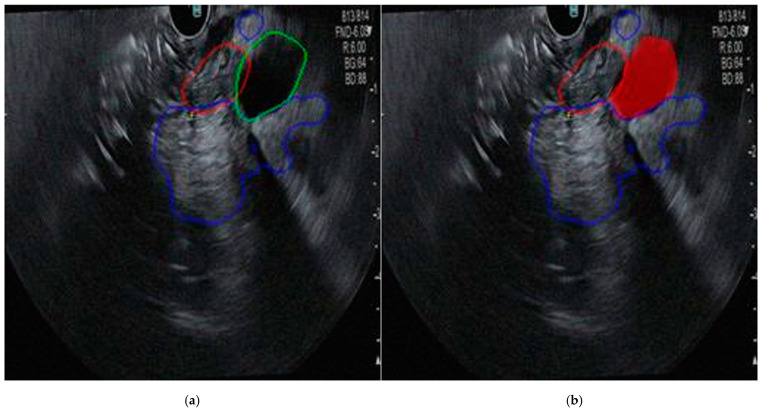
View of the segmented regions, with distinct colors assigned to each organ to enhance visibility and differentiation. (**a**) All contours, including the tumor (red), pancreas (blue), and bile duct (green), are generated automatically by the CNN; (**b**) contours of the tumor (red) and pancreas (blue) are generated automatically by the CNN, while the bile duct (full red) is drawn manually.

In the final phase of the study, a comprehensive tool in MATLAB that integrates all previously implemented models is developed. This application utilizes CNNs for image classification and segmentation of tumors, the pancreas, and the bile duct. The tool features a user-friendly graphical interface that is designed for medical professionals to understand and use easily. This interface, developed using MATLAB’s App Designer, opens with a welcome message and a button labeled “Upload Image” (Figure 9a). This button allows users to select an endoscopic ultrasound image directly from their device. Upon clicking, users can navigate their storage system and choose the image they wish to analyze further. After uploading an image, the application employs the neural network-based classification model to evaluate the presence or absence of cancer. The user interface offers various functionalities essential for analyzing endoscopic ultrasound images, including tumor outline, pancreas outline, biliary duct outline, drawing manual contour, erase contours, and save image. If the segmentation performed by the software is unsatisfactory, users can manually draw the desired contour of any organ in the displayed ultrasound image using the “Draw Manual Contour” button. Activating this feature prompts a guiding message, suggesting that users draw the contour directly on the image by selecting a series of points along the edge of the region of interest. This allows users to manually outline an organ or tumor while preserving automatically generated contours for other regions (Figure 9b). Each organ is outlined in a distinct color, facilitating easy differentiation between regions of interest (Figure 10).

This study has several limitations that should be acknowledged. First, the relatively small dataset may affect the model’s generalizability and its ability to capture the full variability of dCCA presentations in clinical practice. The single-center, retrospective design could also restrict the external validity of our findings, underscoring the need for multi-center studies. Additionally, the model has not been externally validated on independent datasets, which may limit its robustness when applied to different populations or imaging systems. The use of image-based rather than patient-based data separation may introduce bias and lead to overestimation of performance. Furthermore, the model focuses on detecting dCCA, the pancreas, and the bile duct without addressing other biliary pathologies or cholangiocarcinoma subtypes. While incorporating video data could provide additional dynamic information, it has been decided that still images will be used for a number of reasons: (1) Still images ensure consistency and data quality, avoiding variability seen in videos from factors like motion blur; (2) training stability was achieved by controlling uniform image size and format; (3) videos require significantly more computational resources, which was beyond the scope of this study; (4) still images are commonly used in clinical practice, making this approach directly relevant. Expanding to video-based analysis is a valuable future direction. Finally, despite using data augmentation, there remains a risk of overfitting due to the small dataset.

## 5. Conclusions

This study demonstrates the significant potential of AI in enhancing the diagnostic accuracy of EUS for dCCA. A comprehensive MATLAB application that integrates advanced CCNs for the classification and segmentation of EUS images was successfully developed, specifically targeting dCCAs, the pancreas, and the bile duct. The results demonstrate the model’s robust performance, showcasing high accuracy, precision, and reliability in identifying cancerous lesions. These findings highlight the capacity of AI to improve the accuracy and efficiency of diagnosing malignant biliary tumors, potentially reducing the need for invasive procedures and improving patient outcomes. Future research should focus on further validating these results through large-scale, multi-center studies and exploring the integration of AI-assisted EUS into routine clinical practice. This research lays the foundation for prospective studies focused on the identification of pCCA. Additionally, enhancing the model’s capabilities to detect specific features associated with pCCA can aid in earlier diagnosis and better treatment planning. Ultimately, the application serves as a vital tool for medical professionals, facilitating informed decision-making and improving patient outcomes in the diagnosis of cholangiocarcinoma and related pathologies.

## Figures and Tables

**Table 1:** Classification report and segmentation report results for tumor identification.

Classification Report Parameters	Value	Segmentation Report Parameters	Value
Accuracy	0.97	Global Accuracy	0.93
Precision	1	Mean Accuracy	0.74
Sensitivity	0.94	Mean IoU	0.65
Specificity	1	Weighted IoU	0.88

IoU: Intersection over Union.

**Table 2:** Segmentation report results for the pancreas and bile duct.

Pancreatic Segmentation Report	Value	Bile Duct Segmentation Report	Value
Global Accuracy	0.84	Global Accuracy	0.90
Mean Accuracy	0.72	Mean Accuracy	0.63
Mean IoU	0.61	Mean IoU	0.53
Weighted IoU	0.74	Weighted IoU	0.87
Mean Boundary F1 Score	0.49	Mean Boundary F1 Score	0.45

IoU: Intersection over Union.

## Data Availability

The data that support the findings of this study are available on request from the corresponding author, RIO.
